# Insulin‐like growth factor‐2 is a promising candidate for the treatment and prevention of Alzheimer's disease

**DOI:** 10.1111/cns.14160

**Published:** 2023-03-27

**Authors:** G. S. Fitzgerald, T. G. Chuchta, E. C. McNay

**Affiliations:** ^1^ University at Albany Albany New York USA

**Keywords:** alzheimer's disease, neurogenesis, insulin‐like growth factor 2, neurodegenerative disease, dementia, mild cognitive impairment, acetylcholine

## Abstract

Alzheimer's disease (AD) is the most common form of dementia. Current AD treatments slow the rate of cognitive decline, but do not restore lost function. One reason for the low efficacy of current treatments is that they fail to target neurotrophic processes, which are thought to be essential for functional recovery. Bolstering neurotrophic processes may also be a viable strategy for preventative treatment, since structural losses are thought to underlie cognitive decline in AD. The challenge of identifying presymptomatic patients who might benefit from preventative treatment means that any such treatment must meet a high standard of safety and tolerability. The neurotrophic peptide insulin‐like growth factor‐2 (IGF2) is a promising candidate for both treating and preventing AD‐induced cognitive decline. Brain IGF2 expression declines in AD patients. In rodent models of AD, exogenous IGF2 modulates multiple aspects of AD pathology, resulting in (1) improved cognitive function; (2) stimulation of neurogenesis and synaptogenesis; and, (3) neuroprotection against cholinergic dysfunction and beta amyloid‐induced neurotoxicity. Preclinical evidence suggests that IGF2 is likely to be safe and tolerable at therapeutic doses. In the preventative treatment context, the intranasal route of administration is likely to be the preferred method for achieving the therapeutic effect without risking adverse side effects. For patients already experiencing AD dementia, routes of administration that deliver IGF2 directly access the CNS may be necessary. Finally, we discuss several strategies for improving the translational validity of animal models used to study the therapeutic potential of IGF2.

## Graphical abstract

Insulin‐like growth factor 2 (IGF2) is a neurotrophic peptide that is implicated in memory function and declines in Alzheimer's disease (AD). IGF2 may treat AD by opposing neuropathological mechanisms and stimulating neurotrophic processes. Due to its safety and tolerability, IGF2 may also prevent AD in at‐risk individuals. (Created with BioRender.com).

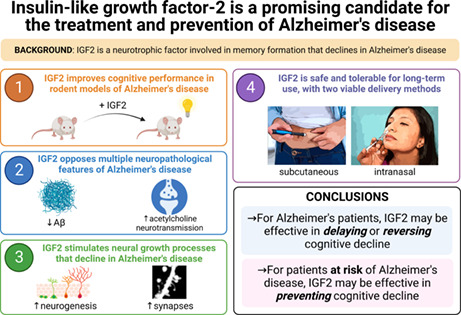

## 1 INTRODUCTION

Alzheimer's disease (AD) is a progressive neurodegenerative disorder that accounts for 60–80% of all dementia cases, and is the seventh leading cause of death globally.^1^ There are currently 20 million AD patients, most of whom are over the age of 65. As demographics shift toward a more aged population, the global prevalence of AD is expected to reach nearly 50 million by 2050.^2^ At present, there are no truly effective treatments for AD. Existing drugs alleviate cognitive symptoms, but fail to significantly alter disease progression. The underwhelming performance of the most recent FDA‐approved AD drug, aducanumab, has highlighted the need for fresh approaches.^3^

An attractive alternative to the existing approaches is *preventative treatment*. While treating those patients who have already manifested AD symptoms is an important objective, it is believed that initiating treatment during the long presymptomatic phase of AD pathogenesis may be a more viable therapeutic strategy. Two of the key challenges to implementing preventative treatment are (1) the relative dearth of empirical research into the pathological mechanisms that occur during the presymptomatic phase and (2) the difficulty of identifying patients at risk of AD. In recent years, prospective studies tracking the changes in neuroimaging biomarkers in patients, as they progress from normal cognition to AD‐associated cognitive impairment, have begun to illuminate the neural changes associated with this transition.^4^, ^5^ Drugs that oppose these early pathological mechanisms may be capable of preventing AD by arresting disease progression and preserving cognitive function. A preventative treatment would likely be a great boon to global health and wellbeing.

There are five compounds currently approved to treat AD. These include three cholinesterase inhibitors (ChEIs)—donepezil, galantamine, and rivastigmine—which potentiate the action of acetylcholine by inhibiting its enzymatic degradation. Because hippocampal acetylcholine is integral to cognitive performance, it is thought that the ChEIs improve cognitive function by rectifying the AD‐associated decline in temporal lobe acetylcholine neurotransmission.^6^, ^7^ Next, there is the partial N‐methyl‐D‐aspartate (NMDA) receptor antagonist memantine, which is thought to attenuate cognitive decline by modulating the excitability of neural circuits, suppressing excitotoxic cell death. Memantine is commonly prescribed in conjunction with a ChEI. Finally, aducanumab is a monoclonal antibody that binds beta amyloid (Aβ), leading to clearance of some Aβ from the brain.^8^ Since amyloid pathology is a major disease process in AD, enhanced disposal of Aβ is presumed to mitigate cognitive impairment by slowing Aβ‐induced neurodegeneration.

None of the current drugs meaningfully halt or reverse cognitive impairment. Instead, these drugs are considered effective based on their ability to slow the *rate* of cognitive decline.^9^, ^10^ This minimal criterion of effectiveness reflects the widespread assumption that progressive cognitive decline in AD is inevitable, with or without treatment.^11^ Furthermore, it is worth noting that the attenuation of cognitive decline provided by current drugs is not necessarily clinically meaningful: with the large sample sizes (>1000 participants per group) marshaled for clinical trials, an effect that is statistically significant may be so small in magnitude as to be clinically unimportant.^12^ Indeed, many of the studies that report statistically significant benefits of these AD drugs fail to reach the conventional threshold for a “minimal clinically relevant improvement”.^12^

Another criterion on which AD treatments can be evaluated is reduced mortality. ChEIs have been shown to modestly extend lifespan in AD patients,^13^, ^14^, ^15^ while neither memantine nor aducanumab is associated with reduced mortality.^10^, ^16^ However, extended lifespan in the absence of functional recovery is unlikely to be viewed as meaningful by patients and caregivers. In economic terms, premature mortality accounts for only 2% of the total cost of dementia in the United States,^17^ with the remaining costs being attributable to the consequences of cognitive deterioration, such as reduced career productivity and the expenses associated with residential care.^17^ Thus, lifespan extension cannot supplant cognitive improvement as the most important benchmark of effectiveness for AD treatments.

In this narrative review, we discuss the therapeutic potential of the neurotrophic peptide insulin‐like growth factor‐2 (IGF2) in AD treatment. In a healthy brain, IGF2 plays a key role in memory formation, neuronal growth, and neuroprotection. Since IGF2 declines in the brains of AD patients, recent studies have explored the possibility that supplemental IGF2 could ameliorate AD pathophysiology. In animal models of AD, exogenous IGF2 has been found to enhance cognition, stimulate neurotrophic processes, and attenuate AD pathophysiology. Such favorable preclinical findings, along with favorable safety and tolerability, make IGF2 an attractive target for AD treatment. Furthermore, the therapeutic utility of IGF2 may also extend to the prevention of AD in patients who have yet to manifest cognitive impairment.

## 2 THE RATIONALE FOR PREVENTATIVE TREATMENT

The comparatively low effectiveness of current AD drugs in arresting cognitive decline is due not only to the failure to target the relevant pathophysiological mechanisms, but also to the relative lateness of drug delivery in the course of AD progression. Current clinical standards recommend drug prescription at the earliest sign of detectable cognitive impairment.^18^ However, in the context of AD pathogenesis, overt cognitive decline is a late event that is preceded by years of neuropathological changes.^19^, ^20^ Thus, by the time cognitive symptoms emerge, brain damage is likely extensive and as such the potential for functional recovery may be significantly reduced.

Moreover, there are compelling reasons to suppose that functional recovery is implausible once the disease has progressed to the point of cognitive impairment. First, there are no documented cases of recovery from AD dementia.^21^ Second, it is perhaps unlikely that a disease that develops over decades could be reversed in the 4–8 years that typically separate initial dementia diagnosis from death.^2^ Third, the endogenous mechanisms that could plausibly mediate functional recovery may be among the damaged mechanisms that give rise to cognitive impairment. Indeed, the milieu created by the neuropathological alterations has been shown to be hostile to the very neurotrophic processes that would be required for regeneration.^22^, ^23^, ^24^, ^25^, ^26^ AD prevention has therefore emerged as an attractive alternative to the challenge of meaningfully treating late‐stage AD. In this review, *preventative treatment* is defined as any treatment that halts the progression from normal cognitive performance into a clinically detectable degree of cognitive impairment.^11^, ^27^, ^28^

### 2.1 Identifying presymptomatic patients for putative preventative treatments

At present, there are no validated models capable of predicting whether a cognitively normal person will go on to develop AD. However, many risk factors have been identified, such as advanced age, APOE genotype, family history of AD, female sex, cardiovascular disease, peripheral insulin resistance, and history of traumatic brain injury.^29^, ^30^ One stratgy for delivering preventative treatment would be administer it to patients at high risk. This rationale has been used to justify the use of statins^31^ and aspirin^32^, ^33^ for preventing cardiovascular disease in patients with significant risk factors and no contraindications. In the context of AD, non‐steroidal anti‐inflammatory drugs (NSAIDs) have already been tested in clinical trials for AD prevention, although no benefit was observed.^34^

### 2.2 Currents drugs are unlikely to be effective preventative treatments

It is unlikely that existing AD drugs would be effective as preventative treatments. To our knowledge, there are no studies to have assessed the ability of existing drugs to prevent the progression of normal cognition to mild cognitive impairment (MCI) or dementia. Currently, the AHEAD Study is recruiting for a clinical trial to test the hypothesis that the anti‐amyloid drug lecanemab can delay or prevent the onset of cognitive impairment in cognitively normal adults with evident brain amyloid accumulation (ClinicalTrials.gov, Identifier: NCT04468659^35^). Notwithstanding this ongoing trial, most studies that purport to use currently approved drugs to “prevent” AD recruit MCI patients and define prevention as fewer patients progressing to clinical dementia. Furthermore, if current drugs were effective in preventative treatment, one would predict that MCI patients would benefit *more* from these drugs than dementia patients since MCI represents an earlier point in disease progression. However, it was found that MCI patients treated with ChEIs showed the same degree of symptom relief as patients with mild or moderate dementia.^36^, ^37^

Based on an analysis of their mechanisms of action, the current AD drugs lack the requisite properties to prevent disease progression. For the ChEIs, inhibiting acetylcholinesterase potentiates acetylcholine action at remaining synapses but has not been shown to rectify the underlying degeneration of cholinergic neurons and their synapses. Among the ChEIs, galantamine is the possible exception, possessing a secondary mechanism of action that may be relevant in preventative treatment. Acting as a positive allosteric modulator of the ɑ4/β2 and ɑ7 nicotinic acetylcholine receptors,^38^, ^39^ galantamine has been shown to enhance neurogenesis in mice.^38^, ^40^ Galantamine's action at the ɑ7 nicotinic acetylcholine receptor may be neuroprotective against Aβ_1–42_ binding at the same receptor.^41^ Despite these additional mechanisms, galantamine is not evidently superior to the other ChEIs in slowing cognitive decline.^15^

Although NMDA receptor dysfunction is a major pathological feature of AD,^42^ it is not clear how memantine would attenuate the various neuropathological mechanisms attributed to the NMDA receptor in AD pathology.^43^, ^44^ Similarly, it is difficult to predict whether aducanumab would be effective if administered earlier in disease progression. Despite shrinking amyloid plaques,^8^ aducanumab has minimal (if any) effect on cognitive outcomes.^3^ However, some studies indicate that the most consequential amyloid pathology occurs early in AD progression, with an elevation in neurotoxic Aβ oligomers.^45^, ^46^ Aducanumab reportedly binds these soluble Aβ species,^8^ suggesting possible efficacy at an earlier stage of disease progression. It is also plausible that averting early amyloid accumulation might prevent later tau pathology, which is thought to be the proximate cause of neuronal death in AD. However, as we will discuss in the next section, aducanumab can likely be excluded from consideration as a preventative treatment due to poor tolerability.

### 2.3 Current AD drugs have poor tolerability

Another obstacle to repurposing the current AD drugs as preventative treatments is their low tolerability. The ChEIs and memantine are prone to adverse side effects, with gastrointestinal discomfort, nausea, and dizziness being especially common; these side effects often prompt patients to discontinue treatment.^13^, ^16^ As a newer drug, the tolerability of aducanumab has not been evaluated as extensively as prior AD drugs. However, aducanumab treatment has a non‐trivial risk of brain edema, requiring periodic “surveillance” MRI scans to detect the neuroimaging biomarkers of this serious adverse event.^10^ This particular risk is elevated in carriers of the APOEε4 allele, who comprise 25% of the population and are at higher risk of developing AD.^47^ It is worth noting that other anti‐amyloid monoclonal antibodies, such as lecanemab, may have binding profiles that improve amyloid clearance while also lowering risk of adverse events.^48^ Treatment with such an antibody may have satisfactory tolerability for use as a preventative treatment.

The adverse side effects elicited by current drugs are often so aversive that AD patients stop taking a drug for which there is no alternative.^13^ For presymptomatic individuals, the tolerability of a preventative treatment is likely to be an even more salient consideration for presymptomatic individuals than for AD patients. For a cognitively unimpaired patient receiving a preventative treatment, any aversive side effects would likely provoke discontinuation, since their experience of the drug would consist entirely of unpleasant symptoms. Thus, a preventative treatment must be highly tolerable to be considered a viable therapeutic.

Because the current AD drugs lack the requisite effectiveness and tolerability for preventative treatment, we need novel treatments that effectively, tolerably, and non‐invasively target pathological processes in early AD, so that cognitive decline can be delayed or prevented.

## 3 NEUROTROPHIC DECLINE IS A MAJOR FEATURE OF AD

As neurodegeneration progresses in AD, pathological mechanisms directly interfere with the operation of existing neurons, culminating in widespread neuronal death.^49^ In addition to this type of direct neurotoxicity, there is a decline of neurotrophic processes such as neurogenesis.^50^ The loss of neurotrophic signaling is implicated in a vicious cycle that is thought to occur in AD progression, wherein early neuropathological changes suppress neurotrophic signaling, which precipitates further neuropathology by removing a source of neuroprotection.^51^ This pathological cascade is thought to contribute to the profound neurodegeneration seen in AD, where as much as 60% of hippocampal neurons may be lost in advanced stages.^52^ Structural atrophy becomes extensive in AD, even before the onset of dementia. Compared to cognitively unimpaired patients, MCI subjects show ~10% lower hippocampal volume,^53^, ^54^ as well as more rapid atrophy in the hippocampus and cerebral cortex.^55^

While the plasticity of neural tissue is remarkable, regeneration of adult neural tissue at a scale commensurate with AD‐induced neurodegeneration has not been reported. Moreover, the mechanism for regenerating lost neurons, neurogenesis, has been shown to decline throughout AD.^50^ In light of the scale and intractability of neurodegeneration in AD, an invasive approach like direct injection of neural stem cells into the brain has been proposed as a therapeutic strategy.^56^ However, even if competent neural precursors could be delivered via a less invasive route of administration, it may still be insufficient to rectify neurodegeneration: in transgenic AD mice, implanted neural precursors often fail to become incorporated because pathological changes have rendered the neural milieu hostile to neuronal maturation.^57^ Taken together, the early manifestation of neurotrophic decline and the implausibility of large‐scale regeneration highlight the importance of preventative treatment strategies in AD.

### 3.1 Neurotrophic signaling is an attractive therapeutic target in AD research

As part of the generalized neurotrophic decline in AD, there is a corresponding decline in *neurotrophic signaling factors*, the biomolecules that mediate growth and reorganization of neurons and synapses. Multiple neurotrophic factors have been implicated in AD pathology, including brain‐derived neurotrophic factor (BDNF)^58^, ^59^ and nerve growth factor (NGF).^60^ Exogenous BDNF and NGF have been shown to exert procognitive and anti‐neurodegenerative effects in rodent models,^61^, ^62^ but these experiments used direct CNS administration (intracranial, intracerebroventricular), which is regarded as too invasive for most human therapies. An invasive route of administration would be especially unsuitable for a preventative therapy, since potential benefits would not outweigh the inherent risk of the procedure. There are currently efforts to replicate the benefits of BDNF and NGF signaling with bioengineered BDNF and NGF analogs that have improved trans‐blood–brain barrier (BBB) transport, as well as BBB‐permeable small molecules that agonize the receptor targets of these neurotrophic factors.^63^ While promising, none of these novel approaches to drug delivery has yet been shown to attenuate cognitive decline or synaptic degeneration in preclinical models of AD.

### 3.2 The neurotrophic factor IGF2 is a promising therapeutic agent

IGF2 is a neurotrophic factor that is decreased in AD patients.^64^, ^65^, ^66^ In recent years, IGF2 has been identified as a modulator of hippocampal cognition that is dysregulated in multiple neurodegenerative disorders, including AD.^67^, ^68^, ^69^, ^70^ IGF2 may have the requisite features of not only a conventional AD treatment, but also a preventative therapy. In rodent models of AD, exogenous IGF2 can in some cases ameliorate cognitive impairments, neuropathological changes,^64^, ^66^ and neurotrophic decline.^71^ In addition to its potential effectiveness, it has, according to the current body of research, favorable safety and tolerability. IGF2 does not require an invasive route of administration. Compared to other neurotrophic factors such as BDNF and NGF, IGF2 has greater BBB permeability, which is associated with greater bioavailability to the brain. This is likely due to IGF2's role as a circulating factor with an endogenous blood‐to‐brain transport mechanism. Moreover, exogenous IGF2 has been shown to recapitulate some of the neurotrophic effects attributed to BDNF^72^ and to increase hippocampal expression of several other neurotrophic factors, including BDNF and NGF.^71^ Taken together, the preclinical data on IGF2 with regard to procognitive and anti‐neurodegenerative effects, favorable safety, and good tolerability suggest that IGF2 may be a promising candidate for the treatment and prevention of AD.

## 4 OVERVIEW OF IGF2

IGF2 is a pleiotropic peptide that belongs to a network of interrelated signaling factors known as the *insulin and insulin‐like growth factor signaling* (IIS) axis. In placental mammals, this axis includes three major ligands: insulin, insulin‐like growth factor‐1 (IGF1), and IGF2. These ligands bind with varying affinity to three main receptors: insulin receptor (IR), IGF1 receptor (IGF1R), and IGF2 receptor (IGF2R). (There are also hybrid receptors that contain an IR and IGF1R subunit). Additionally, IGF1 and IGF2 bind six high‐affinity IGF‐binding proteins (IGFBPs), which regulate the action of IGFs by sequestering them away from receptors. IGFBPs generally inhibit the action of IGFs, but may in some cases enhance their biological action by protecting them from degradation.

In circulation, nearly all IGFs are bound to IGFBPs, with only 0.13% of total IGF2 being “free” to bind receptors.^73^ The half‐life of free IGF2 in circulation is ~10 min, while the half‐life is ~30 min for the IGF2‐IGFBP binary complex, and ~12 h for a ternary complex that also contains the protein acid‐labile subunit *(ALS)*.^74^ However, only the binary complexes are able to cross from capillaries into tissue.^75^ Free IGFs can diffuse across capillary pores to act directly within tissue, although the physiological significance of these free IGFs is not known.^76^ In addition to producing IGFs locally, tissues can dynamically regulate the action of circulating IGFs by secreting enzymes capable of degrading IGFBPs, thereby liberating the IGFs within binary complexes.^77^, ^78^, ^79^, ^80^

The *Igf2* gene has four known gene products: pro‐IGF2 (1–156), two variants of big‐IGF2 (1–87 and 1–104), and fully cleaved (“mature”) IGF2 (1–67) (reviewed in ref. 81). In humans, the predominant *Igf2* product in both circulation and the CNS is fully cleaved IGF2. This pattern is notably different in laboratory rodents, which show CNS expression of fully cleaved IGF2 yet have virtually no fully cleaved IGF2 in the blood.^82^ Rather than signifying the absence of IGF2 bioactivity in the rodent periphery, it is likely that an analogous signaling function is accomplished by the larger gene products, particularly pro‐IGF2.^83^, ^84^, ^85^

IGF2 binds all three receptors in the IIS axis. In the CNS, the neurotrophic effects of IGF2 have been attributed to all three IIS receptors (reviewed in ref. 68). A major physiological function of IGF2 is mitogenic signaling via IGF1R, which promotes growth, differentiation, and survival in multiple tissues. The IR has two alternative splice variants, IR‐A and IR‐B. Of these, IGF2 has nearly five times greater binding affinity for IR‐A.^86^

IR and IGF1R are receptor tyrosine kinases with similar structures and overlapping intracellular signaling pathways. In contrast, IGF2R is a single‐pass transmembrane receptor with no intrinsic kinase domain. Also known as the cation‐independent mannose‐6‐phosphate receptor, IGF2R traffics mannose‐6‐phosphate‐tagged ligands to the endosomes for post‐translational processing or delivery to lysosomes. Consistent with its role in targeting intracellular proteins for lysosomal degradation, the subset of IGF2R that associates with the plasma membrane (PM) internalizes extracellular IGF2 and targets it for lysosomal degradation. Consequently, it was believed that the major function of PM‐bound IGF2R was negative regulation of IGF2 signaling at IGF1R or IR (reviewed in ref. 87). However, recent studies have identified novel signaling properties of IGF2R. Unlike IR and IGF1R, which have well‐defined intracellular signaling pathways, the intracellular mechanisms by which IGF2R transduces its downstream effects are less well characterized. At present, known mechanisms include (1) ERK1/2 phosphorylation followed by recruitment of inhibitory G‐proteins^88^; (2) activation of PKC via a pertussis toxin‐sensitive mechanism ^89^; and, (3) facilitation of cellular autophagy via an unknown mechanism.^90^ While the neurotrophic effects of IGF2 are primarily attributed to increased mitogenic signaling, IGF2 may also modulate brain metabolism; since IGF2 binds IR‐A at a similar affinity to insulin, IGF2 may promote neuronal glucose uptake in the same manner as insulin.^91^, ^92^ Indeed, IGF2 binding at IR‐A is the predominant regulator of glucose uptake during fetal development.^93^, ^94^

### 4.1 IGF2 distribution in the CNS in health and AD

IGF2 is expressed throughout the CNS, acting as a neurotrophic signaling factor in both the parenchyma and cerebrospinal fluid (CSF). Most of the IGF2 in the CNS is assumed to be synthesized within the CNS. This is clear in mice and rats, which have negligible levels of circulating IGF2, although a role for peripherally derived IGF2 in the human brain is a possibility. Among the major cell types in the brain parenchyma, *Igf2* expression is highest in neurons,^95^, ^96^ although microglia also secrete IGF2 in some circumstances.^97^, ^98^ IGF2 and its three receptor targets are highly expressed in the hippocampus, a major site of pathological changes in AD.^99^, ^100^, ^101^, ^102^ Consistent with its important role in synaptic plasticity, IGF2^103^ and its receptors^104^ are highly expressed at synapses. Learning drives synthesis of hippocampal IGF2, promoting memory formation.^90^, ^95^, ^102^, ^105^ There is conflicting evidence about age‐related changes in hippocampal IGF2 in rodent models. Whereas 15‐month‐old mice were reported to have ~20% as much hippocampal IGF2 as 7‐month‐old mice,^64^ there was no difference in total hippocampal IGF2 between 4‐month‐old and 26‐month‐old rats.^103^ In AD, there is dramatic decline in hippocampal IGF2 expression in both human AD patients and transgenic mouse models of AD^64^, ^65^, ^66^ (See Table 1).

**TABLE 1:** Evidence of IGF2 decline in AD.

Species	Brain region	Measurement of IGF2 expression	Mean change in IGF2 expression (relative to comparison group)	Reference
Human	Hippocampus	mRNA	~60% decrease in AD subjects vs. control	Steen et al. (2005)^65^
	Frontal cortex	mRNA	No change in AD subjects vs. control	
	Hypothalamus	mRNA	~60% decrease in AD subjects vs. control	
	Cerebellar cortex	mRNA	No change in AD subjects vs. control	
Human	Frontal cortex	mRNA	Decrease in expression in higher Braak Stages	Rivera et al. (2005)^106^
Human	Prefrontal cortex	mRNA	No change in AD subjects vs. control	Agbemenyah et al. (2014)^107^
APP/PS1 transgenic mouse	Hippocampus	mRNA	No change in transgenic mice vs. wild‐type	
		Protein	No change in transgenic mice vs. wild‐type	
Cynomolgus monkey	Hippocampus	mRNA	No change in an ICV‐streptozotocin‐injected monkey model of AD vs. sham	Lee et al. (2014)^101^
Human	hippocampus	Protein	~60% decrease in AD subjects vs. control	Pascual‐Lucas et al. (2014)^64^
Tg2576 transgenic mouse	Hippocampus	Protein	~80% decrease in tranenic mice vs. wild‐type	
Tg2576 transgenic mouse	Hippocampus	mRNA	~50% decrease in transgenic mice vs. wild‐type	Xia et al. (2019)^66^
	Hippocampus	Protein	~50% decrease in transgenic mice vs. wild‐type	

The IGF2 contained in CSF also exerts neurotrophic effects. The major sources of IGF2 in CSF are the choroid plexus and leptomeninges, which show the highest *Igf2* expression in the entire CNS.^108^, ^109^ In CSF, IGF2 regulates the proliferation of neural precursors on the lining of ventricles via IGF1R; these neural precursors are a source of new neurons in the cerebral cortex.^110^ Whereas the decline in IGF2 in the parenchyma (especially the hippocampus) is both apparent and consequential in AD,^64^ CSF‐IGF2 has yet to be conclusively implicated in AD pathology. CSF‐IGF2 levels are poorly correlated with cognitive impairment or disease progression in AD. In contrast to the reported decline in parenchymal IGF2, several studies have reported a statistically significant *increase* in CSF‐IGF2 in AD patients.^111^, ^112^, ^113^, ^114^ However, the variation in baseline CSF‐IGF2 values across these studies, as well as the magnitude of the reported elevation, makes interpreting these studies challenging.

With respect to IGF2's neurotrophic effects, the parenchyma and CSF are typically regarded as functionally distinct compartments. While it might be presumed that CSF‐derived IGF2 may be recruited to parenchyma that is IGF2‐deficient, evidence of IGF2 interchange between the parenchyma and CSF is lacking. There is bulk movement of substances from parenchyma to the CSF via perivascular flow, which is associated with clearance of waste products.^115^ However, whether this mechanism represents a major route of elimination for parenchymal IGF2 in health or disease is not known. After focal brain lesions, CSF‐derived IGFs are targeted to the damaged parenchyma, presumably to support recovery.^116^ But even if parenchymal uptake of CSF‐derived IGF2 occurs in AD, it is apparently not sufficient to compensate for reductions in hippocampal IGF2 seen in AD patients.^64^, ^65^, ^66^

Many gaps remain in our mechanistic understanding of IGF2 in the brain. A salient obstacle is the complexity of brain IIS signaling, which involves multiple ligands acting on multiple receptors in a cell type‐specific manner. Fortunately, a precise understanding of the relative contribution of each receptor and cell type to the biological action of IGF2 is probably not necessary for evaluating the clinical utility of IGF2. However, since the procognitive and anti‐neurodegenerative effects of IGF2 have been attributed to all three IIS receptors, recognizing receptor‐specific effects may be useful in tailoring IGF2 for use as an AD therapeutic.

### 4.2 The role of circulating IGF2 in neurocognitive function

The blood is another potential source of IGF2 for the CNS. Exogenous IGF2 enters both the parenchyma and CSF.^117^, ^118^ However, it is unclear whether free IGFs are physiologically relevant, given that (as noted) only ~0.13% of IGF2 in circulation is present as free IGF2.^73^ Additionally, at 7.5 kDa, free IGF2 is too large to diffuse across the BBB^119^ and would therefore require a facilitated transport mechanism. Interestingly, the only known mechanism for blood‐to‐brain transport of IGFs involves an IGF being bound to an IGFBP,^80^ perhaps indicating that exogenous IGF2 must complex with an IGFBP before being transported into the brain.

The physiological significance of blood‐to‐brain and blood‐to‐CSF transport of IGF2 is not well understood. As with the IGF2 in CSF, it is plausible that circulating IGF2 could be recruited to the parenchyma in some circumstances. Since such compensatory transport apparently does not occur in AD, it indicates that the mechanism governing blood‐to‐parenchyma IGF2 transport is not activated by low IGF2 signaling in the parenchyma. Instead, blood‐to‐parenchyma IGF2 transport may be driven by local neuronal activity, which stimulates low‐density lipoprotein receptor‐related protein 1 (LRP1)‐mediated transcytosis of circulating IGF‐IGFBP3 complexes across the BBB, followed by the liberation of bound IGFs via matrix metalloproteinase 9 (MMP9)‐mediated cleavage of IGFBP3.^80^, ^120^ While this mechanism was originally described for blood‐to‐brain transport of IGF1, the same mechanism likely transports IGF2, since IGF2 also forms complexes with IGFBP3 (private correspondence with Dr. Torres‐Aleman).

A recent study from Zolov et al.^85^ reports the first example of blood‐to‐CNS transport of *Igf2* gene products. They show that circulating *Igf2* gene products stimulate insulin receptors in the rat retina.^85^ The retina is part of the CNS, and the blood‐retina barrier is structurally and functionally similar to the BBB in how it regulates the movement of proteins across the barrier.^121^ Further research is necessary to determine whether circulating IGF2 is transported across the BBB to supplement endogenous IGF2 signaling in the brain.

The contribution of circulating IGF2 to neural function, including cognition, is poorly understood. There is substantial variation in circulating IGF2 among healthy adults (300–1100 ng/mL^122^), but this natural variation does not appear to correlate with AD status or degree of cognitive impairment.^123^ The IGF2 concentration in CSF is apparently regulated independently of circulating IGF2 levels: in patients with endocrine disorders that result in either constitutively high‐ or low‐serum IGFs, CSF levels of both IGFs remain normal.^124^, ^125^ However, blood‐to‐CSF transport of IGF2 is reportedly reduced in aged sheep.^117^

In summary, the peripheral and CNS concentrations of IGF2 are thought to be distinct pools. Similarly, within the CNS, the parenchyma and CSF are also thought to regulate IGF2 independently. In contrast to the serum and CSF levels, the parenchymal IGF2 levels appear to be the most affected in AD. Despite the fact that circulating IGF2 concentration is not strongly associated with AD risk, understanding the blood‐to‐brain transport mechanisms may be important for developing IGF2 therapeutics that reliably deliver the peptide into the brain.

## 5 IGF2 IS IMPLICATED IN MULTIPLE ASPECTS OF AD PATHOPHYSIOLOGY

### 5.1 IGF2 rescues cognitive impairments in mouse models of AD

As previously discussed, cognitive deterioration is generally regarded as the most devastating aspect of AD. Therefore, it is crucial that a candidate AD treatment suppresses AD‐associated cognitive decline.^126^ IGF2 has been shown to improve hippocampal memory function in transgenic mouse models of AD^64^, ^66^ (see Table 2). Consistent with its role as a modulator of hippocampal cognition,^95^ exogenous IGF2 has been reported to enhance memory formation^90^, ^95^, ^105^, ^129^, ^130^ and to ameliorate age‐related cognitive decline.^103^ The procognitive effects of hippocampal IGF2 have been attributed to both IGF2R^95^, ^105^, ^130^, ^131^ and IGF1R.^127^ It is important to note that the procognitive effects of IGF2 cannot be completely dissociated from its broader neurotrophic role. For example, when intrahippocampal IGF2 enhances the extinction of a contextual fear memory, it does so at least in part by promoting the maturation of 17–19‐day‐old neurons undergoing neurogenesis.^127^

**TABLE 2:** Effects of IGF2 administration in rodent models of AD pathology.

Route of administration (dose, acute vs. chronic administration)	Animal model	Cognitive/Behavioral outcomes	Physiological outcomes	Reference
ICV (50 ng/h, 7 days, chronic)	Mouse (APP.PS1/CHGFP) aged 6 months	n/a	↑ Hippocampal neurogenesis	Mellott et al. (2014)^71^
			↑ Cholinergic neurons in basal forebrain	
			↑ ChAT levels in hippocampus	
			↑ Growth factor expression in hippocampus (BDNF, NGF)	
			↓ Aβ plaques in hippocampus	
Bilateral intrahippocampal microinjection (250 ng IGF2, acute)	Male C57Bl/6 wild‐type mice (3 months old)	Enhanced fear extinction with IGF2 injection	n/a	Agis‐Balboa et al. (2011)^127^
Bilateral intrahippocampal microinjection with viral vector to drive neuronal IGF2 expression (AAV8‐IGF2 construct, acute)	Male C57BL/6 mice (18 months old; female Tg2576 mice (4 months old and 12 months old)	Attenuated cognitive impairment in mice with AAV‐driven IGF2 overexpression	↓ Aβ40, ↓ Aβ42 in prefrontal and parietotemporal cortices	Pascual‐Lucas et al. (2014)^64^
			↑ Dendritic spine density in hippocampus	
Subcutaneous (20 μg/kg, 30 days, daily injection)	Male Wistar rats (103 weeks old)	n/a	↓ Oxidative stress markers in the hippocampus and cortex	Castilla‐Cortazar et al. (2011)^128^

### 5.2 IGF2 alleviates cholinergic dysfunction

Acetylcholine modulates hippocampal cognition, and the decline in acetylcholine neurotransmission is an early pathological event in AD.^6^, ^132^ During a spatial memory task, extracellular acetylcholine levels in the hippocampus nearly double; moreover, the relative increase in acetylcholine correlates with improved task performance.^133^ The underlying causes of reduced acetylcholine neurotransmission in AD are (1) degeneration of cholinergic neurons in the basal forebrain and (2) reduced abundance and activity of choline acetyltransferase (ChAT), the enzyme that synthesizes acetylcholine in the presynaptic terminal. While ChEIs potentiate the action of acetylcholine at remaining synapses, their mechanism fails to address either of these underlying causes. In contrast, IGF2 is reported to preserve cholinergic neurons and potentiate ChAT activity. These properties may afford a therapeutic advantage in treating and preventing cholinergic dysfunction in AD.

IGF2 acutely enhances acetylcholine neurotransmission, albeit by a different mechanism than that of the ChEI drugs. In ex vivo rat brain slices, IGF2 administration evokes acetylcholine release in the hippocampus, striatum, and frontal cortex.^134^ This release is mediated by the IGF2R, which colocalizes with the vesicular acetylcholine transporter (vAchT) in the hippocampus and basal forebrain.^135^ Furthermore, IGF2 modulates the intrinsic responsiveness of basal forebrain cholinergic neurons via an IGF2R‐dependent mechanism; however, it remains unclear how the electrophysiological properties of basal forebrain cholinergic neurons affect acetylcholine neurotransmission in the hippocampus or cerebral cortex.^135^ Further studies are needed to ascertain whether exogenous IGF2 can stimulate hippocampal acetylcholine release in vivo.

In contrast to ChEIs, IGF2 may be able to address the degeneration of cholinergic neurons that occurs in AD pathogenesis. In the transgenic APP/PS1 mouse model of AD, 1 week of chronic intracerebroventricular (ICV) IGF2 infusion enlarged basal forebrain cholinergic neurons while increasing hippocampal ChAT expression.^71^ This was likely mediated by upregulated bone morphogenic factor‐9 (BMP9), a neurotrophic factor that was previously identified as a positive regulator of cholinergic neuron health in the same transgenic AD mouse model.^136^ Whether alone or in combination with a ChEI, IGF2 may relieve cognitive symptoms of AD while also restoring some of the cholinergic signaling infrastructure that is often damaged in AD.

### 5.3 IGF2 ameliorates amyloid pathology

The accumulation of Aβ in the parenchyma is a pathological hallmark of AD, resulting in the formation of amyloid plaques and elevated levels of soluble Aβ. There is evidence to suggest that IGF2 may protect against Aβ‐induced neurotoxicity. In the Tg2576 mouse strain, neuroprotection against elevated Aβ secretion was shown to be mediated by endogenous IGF2 secretion via an IGF1R‐dependent mechanism.^137^

IGF2 administration has been shown to reduce amyloid pathology in mouse models of AD, albeit with routes of administration that would be unsuitable for humans. In APP/PS1 mice, 7 days of chronic ICV IGF2 administration reduced amyloid plaques in the hippocampus.^71^ In the Tg2576 strain, adeno‐associated virus (AAV)‐mediated overexpression of IGF2 in the hippocampus reduced soluble Aβ levels in the prefrontal and parietotemporal cortices, but did not significantly reduce plaque density in the hippocampus itself.^64^ A single intrahippocampal microinjection of IGF2 was sufficient to attenuate hippocampal plaque density and soluble Aβ concentrations 1 week later.^66^ Consistent with a role in Aβ clearance, IGF2 has been shown to reduce non‐amyloid extracellular protein aggregates in a mouse model of Huntington's disease.^138^

In an in vitro model, hippocampal neurons derived from wild‐type mice showed reduced IGF2 expression after being treated with media from cultured neurons derived from the Tg2576 strain, presumably due to the elevated levels of Aβ in the medium.^64^ In the same study, viral vector‐mediated overexpression of IGF2 in hippocampal neurons derived from Tg2576 mice showed near‐total clearance of Aβ from the cell culture medium via an IGF2R‐dependent mechanism. The dual observations of IGF2 attenuating Aβ‐induced damage and Aβ suppressing IGF2 expression are consistent with the hypothesis that AD involves a vicious cycle of escalating pathological changes and neurotrophic decline.^51^

Attenuating dysregulated amyloid pathology is a potential mechanism by which IGF2 can counteract disease progression in early AD. Indeed, interfering with amyloid pathology in the presymptomatic stage may be necessary for a preventative treatment to have a meaningful therapeutic effect. In patients who already display cognitive impairment, aducanumab produces minimal effects on cognitive outcomes despite successfully shrinking amyloid plaques.^3^ Supporting the speculation that targeting Aβ at an earlier stage of disease progression would be more efficacious, Osborne et al.^139^ reported that intrahippocampal administration of an antibody‐like antagonist to oligomeric Aβ rescued cognitive performance in rats fed a high‐fat, high‐sugar diet. Such a diet produces elevated hippocampal Aβ and cognitive dysfunction,^139^ and may therefore be a model that accurately recapitulates early changes that occur in the human brain during AD pathogenesis.

### 5.4 IGF2 and synaptic function

Synaptic degeneration is a major pathological process in AD, with reduced synaptic density in the hippocampus correlating with degree of cognitive impairment.^140^, ^141^, ^142^, ^143^ The density of neurons and synapses in key brain areas has been suggested to be the physiological correlate of *cognitive reserve*, the ability of some patients to resist cognitive decline despite the presence of overt neuropathology.^144^, ^145^ Thus, when evaluating novel therapeutics, the preservation of synapses is likely to be an important benchmark of effectiveness in preclinical research.

Previously, measurements of synaptic density could only be done post‐mortem.^141^ Recently, techniques are being developed to measure synapses in vivo. Using PET, regional synaptic density can be measured using a radiolabeled compound that binds to the ubiquitous synaptic protein *synaptic vesicle glycoprotein 2A* (SV2A).^146^ SV2A‐PET imaging has been recently used in AD patients.^147^, ^148^ Compared to cognitively normal participants, AD patients show reduced SV2A binding in the hippocampus,^147^, ^148^ with this biomarker correlating with impaired episodic memory.^148^

Another avenue by which IGF2 may augment synapses in the hippocampus is by facilitating synaptogenesis. Acting as a neuropeptide, presynaptic IGF2 is necessary for the stabilization of hippocampal synapses formed following synaptic activity.^149^ IGF2 is a downstream effector of the IKK/NF‐κB pathway, which regulates synapse formation and spine maturation in the hippocampus.^131^ In a transgenic mouse model of constitutively downregulated NF‐κB, IGF2 signaling via IGF2R was shown to be necessary and sufficient to rescue synaptic deficits.^131^ In wild‐type mice, overexpression of hippocampal IGF2 resulted in greater spine density on the apical dendrites of CA1 pyramidal neurons.^64^

The synaptic compartment is likely to be an early site of IGF2 dysregulation in AD. In a rat model of age‐related cognitive decline, reduced IGF2 expression in the synaptic compartment was associated with cognitive deficits even as overall IGF2 expression showed no apparent decline.^103^ Since total IGF2 expression eventually declines, dysregulation of synaptic IGF2 may represent an early event in the typical progression of AD.

### 5.5 Hippocampal neurogenesis

A striking manifestation of neurotrophic decline in AD is the reduction in hippocampal neurogenesis that occurs across disease progression.^50^ In neurologically healthy subjects, hippocampal neurogenesis declines with age, but this decline is more dramatic in AD subjects.^50^, ^150^ Rodent studies have indicated a role of hippocampal neurogenesis in many neurological processes that are impaired in related to AD, including memory consolidation^151^, ^152^ and neuroprotection.^153^

While the full significance of adult hippocampal neurogenesis in humans is unclear, an important line of evidence comes from adult patients receiving brain irradiation to treat intracranial tumors. A side effect of this procedure is the death of non‐cancerous cells undergoing cell division, including the neural stem cell precursors that give rise to new neurons. Around half of patients receiving this procedure meet the clinical standard for *radiation‐induced cognitive impairment* (RICD), with symptoms ranging from mild to severe (reviewed in ref. 154). While all RICD studies have the caveat that cognitive decline may be due to cancer progression rather than radiation treatment, these studies suffice to indicate that loss of adult hippocampal neurogenesis is not sufficient to induce profound cognitive impairment. The example of RICD patients may indicate that declining neurogenesis is less consequential than other pathological processes (notably synaptic degeneration) in AD‐associated cognitive decline. This conclusion accords with recent studies that have challenged the claim that adult hippocampal neurogenesis is physiologically significant in humans.^155^, ^156^, ^157^

In the neurogenic zones of the hippocampus, IGF2 acts via IR‐A to maintain the population of neural stem cell precursors.^158^, ^159^ These neural progenitors secrete IGF2 in an autocrine/paracrine manner, although the proximity of these neurogenic zones to the lateral ventricles may allow for IGF2 in the CSF to have an influence.^160^ Seven days of chronic ICV‐IGF2 infusion increased markers of hippocampal neurogenesis in a mouse model of AD,^71^ supporting the idea that IGF2 in the CSF can stimulate neural precursors in the neurogenic zones of the hippocampus. The proliferation of cortical neuron precursors in the ventricular lining is stimulated by IGF2 in CSF via an IGF1R‐dependent mechanism.^110^ In an analysis of gene expression in this population of hippocampal neural stem cells, upregulation of *Igf2* was associated with increased neurogenesis.^161^ In the same study, siRNA‐mediated knockdown of *Igf2* in the hippocampus blunted the proliferation of neural stem cell precursors, while the proliferative action of IGF2 was inhibited by an IGF1R antagonist.

Given the unclear significance of adult hippocampal neurogenesis in human cognition,^157^, ^162^ selecting a putative preventative treatment on the basis of enhanced neurogenesis may not be the optimal approach to preserving cognitive capabilities. It is reasonable to suppose that decline in hippocampal neurogenesis contributes to hippocampal atrophy by subtracting a source of new neurons. While the scale of neurodegeneration in AD is such that repopulation of lost neurons through neurogenesis is implausible, enhancing neurogenesis at an earlier stage of AD pathogenesis may help to mitigate less profound structural losses.

## 6 IGF2 HAS FAVORABLE TOLERABILITY AND SAFETY

Even if IGF2 is effective in enhancing neurotrophic processes and ameliorating AD pathophysiology, its therapeutic utility will also depend on safety and tolerability. As with existing AD drugs, IGF2 will likely require weeks or months of continuous treatment to produce a therapeutic effect. As such, IGF2 must be demonstrated safe and tolerable for long‐term use. The tolerability of IGF2 is especially relevant with regard for its potential use as preventative treatment, since any adverse side effects will seem highly salient as whatever benefits may not be noticeable to the patient.

One of the major factors that influences a drug's tolerability is its viable routes of administration. IGF2 has two convenient routes of administration, subcutaneous and intranasal. In rodent models, IGF2 delivered by either of these routes has been reported to exert neural benefits at concentrations likely to be safe for long‐term use. These routes of administration are suitable for either conventional or preventative AD treatment. Given that the extent of neurological damage and dysfunction is likely greater for AD patients than cognitively normal patients deemed at risk of AD, AD patients may require more invasive routes for delivering IGF2 to the CNS in order to receive a beneficial effect. We discuss several strategies for delivering IGF2 directly to the CNS, concluding with a discussion of gene therapy‐based approaches for driving neural *Igf2* expression.

### 6.1 Routes of administration

#### 6.1.1 Subcutaneous administration

While oral administration is generally considered the most convenient route of administration, the IGF2 peptide is not suitable for oral use because it would be degraded in the gastric fluids.^163^ Subcutaneous injection is likely to be a safe and practical route for systemic IGF2 administration in humans. Indeed, the related peptides insulin and IGF1 are commonly administered subcutaneously for the treatment of diabetes^164^ and growth hormone deficiency,^165^ respectively. Long‐term, repeated subcutaneous administration has already been safely employed in clinical trials of AD therapies.^166^

Several rodent studies have tested the effects of subcutaneously administered IGF2. At 30 μg/kg, a single dose of subcutaneous IGF2 acutely enhances hippocampal memory performance in both mice^167^ and rats,^130^ without affecting metabolic or sensorimotor measures after 30 min, 24 h, or 7 days. There are also data from rats given subcutaneous IGF2 on a chronic or repeated basis. In adult rats, 2 weeks of chronic subcutaneous IGF2 administration, whether with daily injections or constant infusion from an implanted osmotic mini‐pump, does not affect body composition or metabolic measures^168^, ^169^; 30 days of subcutaneous IGF2 reduced markers of oxidative stress in the hippocampus and cortex of aged rats^128^; oxidative stress is a common feature of AD pathology.^170^ However, the impact of chronic IGF2 on cognitive measures is unknown.

There are no published data on the safety or tolerability of IGF2 administration in humans. Despite this lack, comparison to human dosing of the related peptide IGF1 may be of use in predicting the tolerability of IGF2. The primary clinical application for IGF1 is treating growth hormone‐deficient children, to bring their constitutively low IGF1 levels to parity with healthy children at the same developmental stage. This treatment necessitates very high doses of IGF1, so such studies are of limited value when trying to extrapolate the likely side effects of chronic IGF2 administration in an elderly population receiving much lower doses. In a small clinical trial aimed at investigating the potential of IGF1 to prevent bone loss in elderly women, 1 year of twice‐daily subcutaneous injections of IGF1 did not result in a higher rate of adverse side effects than the placebo treatment.^171^ However, a comparable study design that employed a higher dose of IGF1 did indeed cause a higher rate of adverse side effects, including joint pain, edema, nausea, and headaches.^172^

A potential issue with the subcutaneous route of administration is that AD patients may have impaired blood‐to‐brain IGF2 transport, which would compromise effectiveness by limiting bioavailability to the brain. The only characterized mechanism for blood‐to‐brain IGF transport relies on neurovascular coupling,^80^ a process known to be dysregulated in AD.^173^ Therefore, the bioavailability of subcutaneously administered IGF2 to the brain may be enhanced by concomitant stimulation of neuronal activity. While the implications of this inference have yet to be studied, some speculation is warranted. In cognitively normal subjects, or those with moderate levels of cognitive decline, IGF2 may be administered alongside a cognitively stimulating activity such as crossword puzzle or video game. In AD patients with more severe dementia, engagement with a cognitively stimulating activity may be impossible; as such, concurrent use of a non‐invasive brain stimulation method such as transcranial magnetic stimulation or direct current stimulation may be more efficacious.^174^

#### 6.1.2 Intranasal administration

Intranasal administration is a non‐invasive means of delivering compounds to the brain that may circumvent some of the potential issues with subcutaneous administration, particularly the concerns about bioavailability to the brain. Intranasally administered substances bypass the BBB by diffusing across the olfactory epithelium and cribriform plate to reach the brain parenchyma.^175^ Once in the brain, the substances are distributed deeper into the brain along the olfactory and trigeminal tracts, and then outward into the parenchyma via perivascular spaces.^176^

In the only published study of intranasal IGF2, Pardo et al.^72^ reported that intranasal IGF2 rescued spatial memory deficits in mice with siRNA‐induced BDNF deficiency.^72^ Acute intranasal administration of other neurotrophic factors has been explored in rodent AD models. Intranasal BDNF administration rescued memory deficits in AD11 mice, a transgenic AD model with NGF deficiency.^177^ Intranasal NGF improved memory deficits in both AD11 and APP/PS1 transgenic AD mice.^178^, ^179^ Intranasal administration of plasma enriched in growth factors enhanced neurogenesis in the APP/PS1 transgenic mice.^180^

To date, no human or animal studies have investigated long‐term intranasal administration to treat AD using any neurotrophic factor other than insulin. Human studies of intranasal insulin can inform our assessment of the feasibility of long‐term intranasal IGF2 administration. Intranasal insulin has been extensively investigated as a potential AD therapy in humans,^181^ and several studies have reported procognitive and anti‐amyloidogenic effects in cognitively impaired older patients (reviewed in ref. 182). While 12 months of daily intranasal insulin administration failed to delay the progression of cognitive decline in patients with MCI or AD dementia,^183^ the chronic intranasal administration procedure was found to be both safe and tolerable for this population of older patients. The median adherence rate exceeded 90% for the more‐preferred intranasal device.^183^ This pattern of data supports the idea that long‐term intranasal administration of IGF2 is feasible for the population of patients who either have, or are at risk of, AD.

In rats, both insulin and IGF1 show widespread distribution in the brain after intranasal administration.^184^, ^185^ Because distribution of intranasally administered substances is mainly determined by the size of the administered compound,^186^ the distribution of intranasal IGF2 is presumed to be similar to that of IGF1. Based on functional neuroimaging studies of intranasal insulin in humans,^187^, ^188^, ^189^ the distribution of intranasally administered substances is sufficiently widespread to produce detectable neurological effects. Fortuitously, the site at which intranasally administered substances cross over into the brain (the cribriform plate) is located relatively near key structures implicated in AD, including the hippocampus, frontal cortex, and basal forebrain cholinergic nuclei, meaning that compounds would not need to traverse the entire length of the brain to reach these target areas.

#### 6.1.3 Direct administration of IGF2 into the CNS

Although systemic routes of administering IGF2 have shown promise in preclinical studies of AD mouse models, administering IGF2 directly into the CNS may be necessary to achieve functional recovery in human AD patients because the mechanisms regulating blood‐to‐brain transport of IGFs may be compromised in AD. Most in vivo studies examining the neurobiological effects of exogenous IGF2 have used intracranial or ICV routes of administration, so it remains possible that such routes will be required to achieve the same benefit in humans. While such procedures appear to be well‐tolerated in rodents, the neurosurgery that would be required for analogous procedures in humans is liable to be riskier, as human AD patients are disproportionately elderly and frail.^190^ The actual risks of intracranial or ICV administration are difficult to assess, but the general scope of the risks may be inferred from the several clinical trials of AD patients receiving deep brain stimulation (DBS), which involves implantation of electrodes into the fornix, nucleus basalis of Meynert, or ventral capsule. Luo et al.^191^ found a low rate of adverse events following DBS in AD patients,^191^ but it should be noted that the electrode implantation is a single procedure while long‐term IGF2 infusion would involve multiple procedures. More procedures increase the overall risk of complications, potentially undermining the safety of neurosurgical approaches to IGF2 delivery.

A less invasive alternative to intracranial and ICV administration is intrathecal administration, wherein substances are injected into the CSF at the base of the spinal column. While less invasive than neurosurgical approaches, it remains unclear whether peptides in the CSF actually penetrate the parenchyma. ICV‐IGF1 is rapidly cleared from the CSF, resulting in limited distribution throughout the brain.^192^, ^193^ Since IGF1 and IGF2 are of similar size, IGF2 in the CSF is therefore unlikely to diffuse from the CSF into the parenchyma. As such, delivery of IGF2 into spinal CSF may fail to produce the therapeutic benefits attributed to increased IGF2 action in the hippocampus.^95^, ^129^

Notwithstanding the sparse evidence that IGF2 delivered to the CSF enters the parenchyma, Mellott et al.^71^ reported that chronic ICV‐IGF2 reduced amyloid markers and increased ChAT expression in the hippocampus of APP/PS1 transgenic mice.^71^ This would seem to indicate that CSF‐derived IGF2 reaches the parenchyma. A study by Kan et al.^194^ offers evidence that may explain how ICV‐IGF2 can be efficacious despite the poor penetrance of CSF IGF2 into the parenchyma. In a transgenic mouse model of mucopolysaccharidosis type IIIB, a lysosomal storage disorder characterized by lack of the lysosomal enzyme α–N‐acetylglucosaminidase (NAGLU), an IGF2‐NAGLU fusion peptide was chronically infused into the CSF four times over a 2‐week period. The IGF2 component of the engineered peptide bound to IGF2R, delivering NAGLU to the lysosomes. The fusion peptide distributed widely throughout the brain, not simply in regions adjacent to the CSF, as one would expect if the IGF2‐NAGLU peptide moved directly from CSF into parenchyma.^194^ Instead, the fusion peptide that reached the parenchyma may have exited the CSF and the re‐entered the brain via blood‐to‐parenchyma transport. It is clear that the fusion peptide entered circulation because NAGLU was observed to accumulate in the liver. It is thus unclear whether IGF2 administered into the CSF had an effect beyond what that which would be obtained from a systemic route of administration. Further research is needed to determine whether accessing the CSF affords a distinct therapeutic advantage that outweighs the risks of such an invasive procedure.

#### 6.1.4 Gene therapy‐based approaches

Gene therapy is an alternative method for delivering IGF2 to the brain that has the potential to confer durable therapeutic effects without the need for multiple neurosurgical procedures.^195^ Pascual‐Lucas et al.^64^ reported that a single intrahippocampal dose of an AAV‐IGF2 construct produced a 20‐fold increase in hippocampal *Igf2* expression that persisted for up to 8 months in Tg2576 mice, rescuing synaptic deficits and hippocampal memory impairments.^64^ If a single neurosurgical procedure could confer a persistent effects of this magnitude to AD patients, the potential benefits of gene therapy might outweigh the risks of neurosurgery. Intracranial delivery of AAV vectors is an approach that is already being tested for AD and other neurodegenerative diseases, demonstrating a favorable safety profile.^195^ Driving IGF2 expression is consistent with previous gene therapy‐based approaches: in humans and non‐human primates, viral vectors have been used to increase expression of neurotrophic factors such as BDNF^196^ and NGF.^197^, ^198^, ^199^

While most gene therapy studies employ intracranial administration, there is ongoing preclinical work in rats to optimize intrathecal administration of AAV to drive gene expression in the brain.^200^ Intrathecal administration is not only less invasive than intracranial administration, but may also enable more widespread distribution of viral vectors throughout the CNS. However, as with IGF2 itself, penetration of viral particles from the CSF into the parenchyma may be an obstacle to effectiveness. Intracranial administration allows for more precise targeting of specific brain structures, such as the hippocampus. In the future, CRISPR‐Cas9 methods may enable more precise and efficient targeting of particular cellular populations.^201^ While further optimization is necessary, gene therapy‐based approaches for driving neural IGF2 expression may be attractive for AD patients who do not show functional recovery from systemic routes of administration.

### 6.2 Potential risks of IGF2 administration: hypoglycemia and cancer

#### 6.2.1 Hypoglycemia

Through binding at the insulin receptor, IGF2 can stimulate cellular glucose uptake.^202^ Consequently, systemically administered IGF2 has the potential to cause hypoglycemia. IGF2‐induced hypoglycemia is seen in several cancers (e.g. Wilms's tumor, non‐islet cell tumor hypoglycemia) due to hyper‐secretion of IGF2 by the tumor itself.^81^, ^203^ In such patients, serum IGF2 levels are as much as fourfold higher than normal.^204^ The hypoglycemia risk of exogenous IGF2 is primarily influenced by two factors: dose and route of administration. Whereas an intravenous dose of 1 mg/kg IGF2 induced hypoglycemia in mice,^87^ a subcutaneous dose of 30 μg/kg (which was sufficient to cause acute cognitive enhancement) had no effect on blood glucose concentration in either mice^167^ or rats.^130^ Compared to insulin, IGF2 is ~1% as effective at stimulating glucose disposal.^130^, ^202^ Thus, therapeutic doses of subcutaneous IGF2 sufficient to confer neural benefits are not expected to cause hypoglycemia. The hypoglycemia risk of intranasally administered IGF2 is likely to be even lower, since a negligible amount of the intranasally administered compound enters general circulation: whereas 40 I.U. subcutaneous insulin would provoke hypoglycemia in humans, this same dose of intranasal insulin was not associated with acute or chronic hypoglycemia.^183^ Because IGF2 is less effective than insulin in stimulating glucose uptake, even a large dose of intranasal IGF2 would be unlikely to cause hypoglycemia.

#### 6.2.2 Cancer

Another potential risk of therapeutic IGF2 is increased cancer incidence. Because proto‐oncogenic cells often upregulate *Igf2* in order to evade apoptosis,^205^ it is plausible that exogenous IGF2 might increase the risk of a precancerous tissue surviving to become tumorous.^87^ In transgenic mice that overexpress IGF2 in specific tissues, tumor formation tends to increase in those tissues where IGF2 is overexpressed.^206^, ^207^, ^208^ However, such models do not give a clear indication of how systemic administration of exogenous IGF2 would influence cancer risk, as most tissues also secrete IGF2 for use in paracrine and autocrine signaling. The exception is liver tissue, which secretes the majority of circulating IGF2. In a transgenic mouse model with liver‐specific IGF2 overexpression, a 20‐ to 30‐fold increase in circulating IGF2 (relative to wild‐type mice) did not lead to a widespread tumor formation. Rather, spontaneous tumor formation was largely restricted to the liver itself, consistent with the other mouse models with tissue‐specific IGF2 overexpression.^209^ Because these studies involved a constitutive elevation in serum IGF2 far beyond what would occur with a therapeutic dose of subcutaneous IGF2, we infer that a therapeutic dose of IGF2 would be unlikely to trigger oncogenesis/tumorgenesis. The cancer risk of chronic intranasal IGF2 administration is likely to be low, as well; 1 year of daily intranasal administration of insulin, which is also a mitogenic peptide, did not increase cancer incidence in a large clinical trial.^183^ While the available findings suggest that exogenous IGF2 has a low oncogenic risk at therapeutic doses, the actual risk must be determined through clinical studies.

### 6.3 IGF2 analogs and IGF2‐IGFBP complexes may enhance therapeutic effects and avert tolerance

In addition to wild‐type IGF2, several IGF2 analogs have been developed. These analogs show unique binding properties, such as (1) selectivity in binding to particular receptors, and (2) reduced binding to IGFBPs. Such analogs may offer therapeutic advantages over wild‐type IGF2, such as higher potency, fewer off‐target effects, and reduced risk of physiological tolerance. As previously discussed, the neurocognitive benefits of IGF2 have been attributed to all three IIS receptors (IGF2R, IGF1R, and IR). In order to reproduce the full range of IGF2's effects, wild‐type IGF2 should be the default therapeutic option. If future studies indicate that binding at a specific receptor produces undesirable effects, these receptor‐selective analogs may potentially be used to provide the beneficial dimension of IGF2 signaling without the drawbacks.^158^, ^210^

For systemically administered IGF2, it is unclear whether free IGF2 or IGF2‐IGFBP complexes are more bioavailable to the brain. If free IGF2 readily crosses into the CNS,^118^, ^130^ IGFBP‐evading analogs (such as Des[1,6]‐IGF2 and Arg[3]‐IGF2) would be expected to have better bioavailability to the brain than wild‐type IGF2. If, however, the principal mechanism of blood‐to‐brain IGF2 transport involves uptake of IGF2‐IGFBP3 complexes,^80^ administering free IGF2 would be less advantageous than administering IGF2‐IGFBP3 complexes. While the neurocognitive effects of exogenous IGF2‐IGFBP complexes have yet to be studied, subcutaneous administration of IGF2‐IGBP2 complexes was well tolerated in a rat model.^168^

In long‐term drug treatment, tolerance is a common occurrence. While IGF2 tolerance has not been reported, tolerance to insulin (i.e. insulin resistance) underlies Type 2 diabetes and is commonly seen in the AD brain.^211^ As such, the possibility of IGF2 tolerance—whether systemic or central—merits discussion. One possible mechanism for systemic IGF2 tolerance is upregulation of circulating IGFBPs.^212^ In hypophysectomized rats with constitutively low IGF1, 28 days of subcutaneous IGF1 administration increased IGFBP3 expression.^213^ This is consistent with a homeostatic model in which hepatic IGF signaling triggers IGFBP expression as a means of maintaining a setpoint of bioactivity for circulating IGFs. Similarly, upregulation of IGFBPs may be capable of causing IGF2 tolerance within the CNS.^127^ In addition to the six high‐affinity IGFBPs, the IGFBP7 is a lower affinity binding partner.^214^, ^215^ IGFBP7 is upregulated in the prefrontal cortex of AD patients, perhaps indicating that the AD‐associated reduction in brain IGF2 signaling may stem not only from reduced expression but also from increased sequestration of extracellular IGF2.^107^, ^127^ In addition to binding IGF2 in the typical manner of IGFBPs, IGFBP7 may also suppress IGF2 action by directly antagonizing IGF1R.^216^ If tolerance to exogenous IGF2 administration is observed, and it is shown to be caused by upregulated IGFBPs, use of an IGF2 analog with reduced IGFBP binding may be warranted.

## 7 TRANSLATIONAL CONSIDERATIONS

### 7.1 Species differences in IGF2 regulation

Peripheral IGF2 regulation differs substantially between humans and laboratory rodents. Whereas human blood contains abundant IGF2, mice and rats show negligible amounts of circulating IGF2.^82^ Consequently, it is difficult to investigate key mechanisms in rodent models, such as (1) the role of endogenous circulating IGF2 in neurocognitive function and (2) the putative blood‐to‐brain transport mechanism for circulating IGF2. Unless resolved, these species differences undermine the translational validity of rodent models of IGF2 treatment.

The absence of circulating IGF2 in rodents likely does not indicate the absence of IGF2‐like bioactivity, but instead the secretion of a different *Igf2* gene product. Whereas IGF2 is the major *Igf2* gene product in human serum, rodent serum contains 95% in the form of the precursor pro‐IGF2.^83^, ^84^ Qui et al.^84^ reported that the concentration of circulating pro‐IGF2 (26 kDa) in adult rats is ~33 nM, or 858 ng/mL.^84^ This value is comparable to the typical human values for serum IGF2, which can range from 300 to 1100 ng/mL.^122^ Pro‐IGF2 is reported to have binding affinity comparable to IGF2 at receptors and IGFBPs.^83^, ^217^ The existence of these bioactive *Igf2* gene products in rodent serum was previously overlooked because these precursors were not immunoreactive with common IGF2 antibodies.^84^ Interestingly, the source of circulating IGF2/pro‐IGF2 may differ across species. Whereas the liver is the predominant source of circulating IGF2 in humans, liver *Igf2* synthesis is reported to be virtually zero in adult mice and rats.^82^, ^102^, ^108^, ^218^ Zolov et al.^85^ report that circulating *Igf2* products directly subserve retinal insulin signaling, suggesting an alternative, non‐hepatic origin of circulating *Igf2* products.^85^ Further studies into the sources and functions of circulating *Igf2* products in rodents are needed, so that we can best utilize rodent models to produce translationally valid insights.

### 7.2 Dietary choline deficiency may distort preclinical IGF2 research

The nutritional content of the laboratory diet is an underappreciated variable in preclinical research.^219^, ^220^ Choline is an essential nutrient that serves as a precursor to membrane phospholipids and acetylcholine. Choline deficiency during gestational development has been implicated in neurocognitive impairments in the offspring,^221^, ^222^ prompting the FDA to recommend increased choline intake for pregnant women (reviewed in ref. 223). The role of prenatal choline in neurological development has been further explored in rodent models, where prenatal choline supplementation has been reported to enhance cognitive performance.^224^ Napoli et al.^225^ compared rats whose mothers were fed either standard laboratory chow, choline‐supplemented chow, or choline‐deficient chow. At 3 months old, rats whose mothers were fed a choline‐supplemented diet had four times more IGF2 in the hippocampus, and 2.5 times more IGF2 in the frontal cortex, than rats whose mothers were fed the standard laboratory chow.^225^ Maternal choline supplementation also resulted in increased IGF2R expression in the hippocampus, frontal cortex, and medial septum. Consistent with earlier studies demonstrating the role of IGF2 in acetylcholine release,^135^ rats whose mothers received supplemental choline showed elevated acetylcholine release in the hippocampus and frontal cortex. In APP/PS1 transgenic AD mice, perinatal choline supplementation resulted in significantly more hippocampal IGF2 in 9‐month‐old mice.^226^ While these studies did not assess cognitive outcomes, another study that employed a similar paradigm reported that prenatal choline supplementation improved performance in the Morris water maze,^227^ a hippocampal memory task that is often used to assess cognitive function in rodent models.^66^, ^107^, ^131^

Although the choline contents of the aforementioned diet conditions are described as “enriched,” “standard,” and “deprived,” there is scarce reason to believe that the “standard” diet reflects a normative baseline. As a historical matter, the standard laboratory diet was optimized for basic health and fecundity,^228^ with no consideration given to the cognitive capabilities of the offspring. Developing a rational alternative to the standard laboratory diet is challenging, since—to our knowledge—there are no data on the choline intake of wild rodents to serve as an ecologically valid baseline.

If, as the literature indicates, prenatal choline deprivation results in brain‐wide reductions in IGF2 content, this may bias research toward detecting an effect of exogenous IGF2. Since such animals are nearer to the theoretical lower limit of brain IGF2 content, a given dose of exogenous IGF2 would produce a larger fold‐change in IGF2 levels compared to an animal with adequate prenatal choline exposure. This is a challenge to translational validity because the majority of the population in developed nations can be assumed to have had sufficient prenatal choline. To enhance translational validity, the choline content in the maternal diet of laboratory rodents should be optimized to better reflect the normative state of humans in developed nations.^221^ Publications should also report the nutritional content of the maternal diet. Further research is needed to determine whether adequate prenatal choline exposure is sufficient to block the procognitive and anti‐neurodegenerative effects of exogenous IGF2 in laboratory rodents.

### 7.3 Preclinical models of sporadic AD

AD is classified into two distinct etiologies: Familial AD (fAD), caused by the presence of disease alleles in genes that are directly involved with amyloid or tau pathophysiology, and sporadic AD (sAD) that is likely the result of multiple genetic and environmental risk factors. Although sAD accounts for ~90% of all AD cases,^2^ the most common preclinical AD models are transgenic mice recapitulating fAD mutations.^229^ Since the pathogenesis of fAD is not representative of sAD, the translational validity of findings based on fAD models to the majority of AD patients is questionable.^230^ A major limitation of fAD models is that they are less suitable for studying preventative treatments. Whereas the risk of sAD is thought to be reducable through adoption of healthy lifestyle choices,^231^, ^232^ fAD diagnosis is guaranteed if one possesses a disease‐causing genotype. Inasmuch as animal models of fAD also recapitulate this inevitable aspect disease progression, it would be impossible to investigate the ability of a treatment to prevent AD. Given the importance of preventative treatments, preclinical models of sAD will be critical for assessing the preventative potential of novel therapies (such as IGF2). One such rodent model of sAD involves feeding rodents a high‐fat, high‐sugar diet. Such a diet exacerbates known metabolic risk factors for AD, while causing AD‐like hippocampal dysfunction.^91^, ^139^, ^233^

## 8 IGF2 IN OTHER NEURAL DISORDERS

While this review has highlighted the therapeutic potential of IGF2 in AD, IGF2 is also being explored as a treatment for other neural disorders. IGF2 has been reported to improve neurocognitive functioning in patients with autism spectrum disorder,^234^ Huntington's Disease,^138^ amyotrophic lateral sclerosis (ALS),^235^ and Angelman Syndrome.^167^ IGF2 has also been found to rescue age‐related cognitive decline in rats.^103^

## 9 CONCLUSION

IGF2 dysregulation is implicated in multiple neuropathological aspects of AD. In preclinical AD models, exogenous IGF2 improves cognition and ameliorates neuropathology. These mechanisms of action may retard cognitive decline better than current AD drugs, and even promote functional recovery. Based on the available research, long‐term treatment with IGF2 is predicted to be safe and tolerable. Such a favorable therapeutic profile suggests a potential use for IGF2 in treating AD patients alongside existing drugs. Additionally, IGF2 may have value as a preventative AD treatment given to cognitively normal patients who are classified as being at elevated risk of developing AD. The subcutaneous and intranasal routes appear to be the most viable means of administering IGF2. Human trials will likely be necessary to determine which route of administration is optimal. The therapeutic potential of IGF2 is further enhanced by the existence of synthetic analogs with modified potency and receptor‐binding profiles. For treatment of severe AD, gene therapy‐based approaches that drive brain IGF2 expression may be a viable therapeutic option. Future studies should investigate the preventative potential of long‐term IGF2 administration in an animal model of sporadic AD.

## FUNDING INFORMATION

Work on this manuscript was supported by a grant from the NIA (R01AG050598, awarded to ECM) and by an award from the Merlin Foundation to ECM.

## CONFLICT OF INTEREST STATEMENT

The authors declare no competing interests for this work.

## Data Availability

Data sharing is not applicable to this article as no new data were created or analyzed in this study.
